# *In vitro* activity of new combinations of β-lactam and β-lactamase inhibitors against the *Mycobacterium tuberculosis* complex

**DOI:** 10.1128/spectrum.01781-23

**Published:** 2023-09-22

**Authors:** Elin Economou Lundeberg, Viktoria Andersson, Maria Wijkander, Ramona Groenheit, Mikael Mansjö, Jim Werngren, Teresa Cortes, Ivan Barilar, Stefan Niemann, Matthias Merker, Claudio U. Köser, Lina Davies Forsman

**Affiliations:** 1 Department of Infectious Diseases, Central Hospital of Kristianstad, Kristianstad, Sweden; 2 Department of Infectious Diseases, Karolinska University Hospital, Stockholm, Sweden; 3 Department of Microbiology, Public Health Agency of Sweden, Stockholm, Sweden; 4 Pathogen Gene Regulation Unit, Biomedicine Institute of Valencia (IBV), CSIC, Valencia, Spain; 5 Molecular and Experimental Mycobacteriology, Research Center Borstel, Borstel, Germany; 6 German Center for Infection Research, Partner site Hamburg-Lübeck-Borstel-Riems, Borstel, Germany; 7 Evolution of the Resistome, Research Center Borstel, Borstel, Germany; 8 Department of Genetics, University of Cambridge, Cambridge, United Kingdom; 9 Department of Medicine, Division of Infectious Diseases, Karolinska Institutet, Solna, Sweden; University of Southern California, Duarte, California, USA

**Keywords:** β-lactams, β-lactamases, tebipenem, meropenem, clavulanic acid, minimum inhibitory concentrations, vaborbactam, drug resistance mechanisms, BlaC

## Abstract

As meropenem-clavulanic acid is recommended for the treatment of drug-resistant tuberculosis, the repurposing of new carbapenem combinations may provide new treatment options, including oral alternatives. Therefore, we studied the *in vitro* activities of meropenem-vaborbactam, meropenem-clavulanic acid, and tebipenem-clavulanic acid. One hundred nine *Mycobacterium tuberculosis* complex (MTBC) clinical isolates were tested, of which 69 were pan-susceptible and the remaining pyrazinamide- or multidrug-resistant. Broth microdilution MICs were determined using the EUCAST reference method. Meropenem and tebipenem were tested individually and in combination with vaborbactam 8 mg/L and clavulanic-acid 2 and 4 mg/L, respectively. Whole-genome sequencing was performed to explore resistance mechanisms. Clavulanic acid lowered the modal tebipenem MIC approximately 16-fold (from 16 to 1 mg/L). The modal meropenem MIC was reduced twofold by vaborbactam compared with an approximately eightfold decrease by clavulanic acid. The only previously described high-confidence carbapenem resistance mutation, *crfA* T62A, was shared by a subgroup of lineage 4.3.4.1 isolates and did not correlate with elevated MICs. The presence of a β-lactamase inhibitor reduced the MTBC MICs of tebipenem and meropenem. The resulting MIC distribution was lowest for the orally available drugs tebipenem-clavulanic acid. Whether this *in vitro* activity translates to similar or greater clinical efficacy of tebipenem-clavulanic acid compared with the currently WHO-endorsed meropenem-clavulanic acid requires clinical studies.

**IMPORTANCE:**

Repurposing of already approved antibiotics, such as β-lactams in combination with β-lactamase inhibitors, may provide new treatment alternatives for drug-resistant tuberculosis. Meropenem-clavulanic acid was more active *in vitro* compared to meropenem-vaborbactam. Notably, tebipenem-clavulanic acid showed even better activity, raising the potential of an all-oral treatment option. Clinical data are needed to investigate whether the better *in vitro* activity of tebipenem-clavulanic acid correlates with greater clinical efficacy compared with the currently WHO-endorsed meropenem-clavulanic acid.

## INTRODUCTION

Tuberculosis is one of the leading bacterial causes of death globally (1). The rise of *Mycobacterium tuberculosis* complex (MTBC) strains resistant to traditional antimicrobials has highlighted the need to develop new drugs and to repurpose older agents, especially as many new treatment options are expensive and not globally accessible. Treating multidrug-resistant tuberculosis (MDR-TB) is complicated by having to administer multiple drugs with many side effects for a long period of time. β-lactam antibiotics are available and affordable alternatives with extensive clinical experience and fewer side effects (2 – 4). β-lactam antibiotics prevent cell wall synthesis by inactivating D,D-transpeptidases which finalize the cross-linking of peptidoglycans, a major component of the bacterial cell wall. In MTBC, another enzyme, L,D-transpeptidase, is also involved in the peptidoglycan construction (5, 6) and it has been suggested that both enzymes need to be inactivated to stop cell wall synthesis in MTBC (7).

Unfortunately, MTBC is intrinsically resistant against many β-lactams (8). For example, MTBC has an Ambler class A β-lactamase (BlaC) that hydrolyzes penicillins, cephalosporins, and to some extent also carbapenems (9, 10). Indeed, MTBC mutants with inactive *blaC* have been shown to be more susceptible to β-lactams (11). In addition, other proteins, such as Rv0406, Rv3677 (12), and CrfA (13), might also exert β-lactamase activity. However, carbapenems have shown to be more active than other β-lactams as they inhibit L,D-transpeptidases (7) and are more slowly hydrolyzed by BlaC (10). Furthermore, by inactivating the BlaC with a β-lactamase inhibitor, multiple studies have demonstrated *in vitro* activity of carbapenems against MTBC (10, 14 – 18). Indeed, meropenem-clavulanic acid is currently classified by WHO as a Group C treatment option for MDR-TB (19).

In recent years, several new combinations of carbapenems and β-lactamase inhibitors have been developed to combat drug-resistant Gram-negative bacteria, such as meropenem-vaborbactam. Furthermore, there is an orally available carbapenem, tebipenem, which has recently shown promising results in a large trial of complicated urinary tract infections (20). The only orally available β-lactamase inhibitor is clavulanic acid, as part of the amoxicillin-clavulanic acid combination. The repurposing of these new, already approved, drugs might also provide new treatment alternatives for MDR-TB. Therefore, we studied *in vitro* activities of meropenem-vaborbactam, meropenem-clavulanic acid, and tebipenem-clavulanic acid by minimum inhibitory concentration (MIC) testing of clinical MTBC isolates. Additionally, the impact of potential resistance mechanisms on MICs was explored by analyzing whole-genome sequencing (WGS) data.

## RESULTS

### Overview of *in vitro* activity of β-lactams–β-lactamase inhibitor combinations

**Fig 1:**
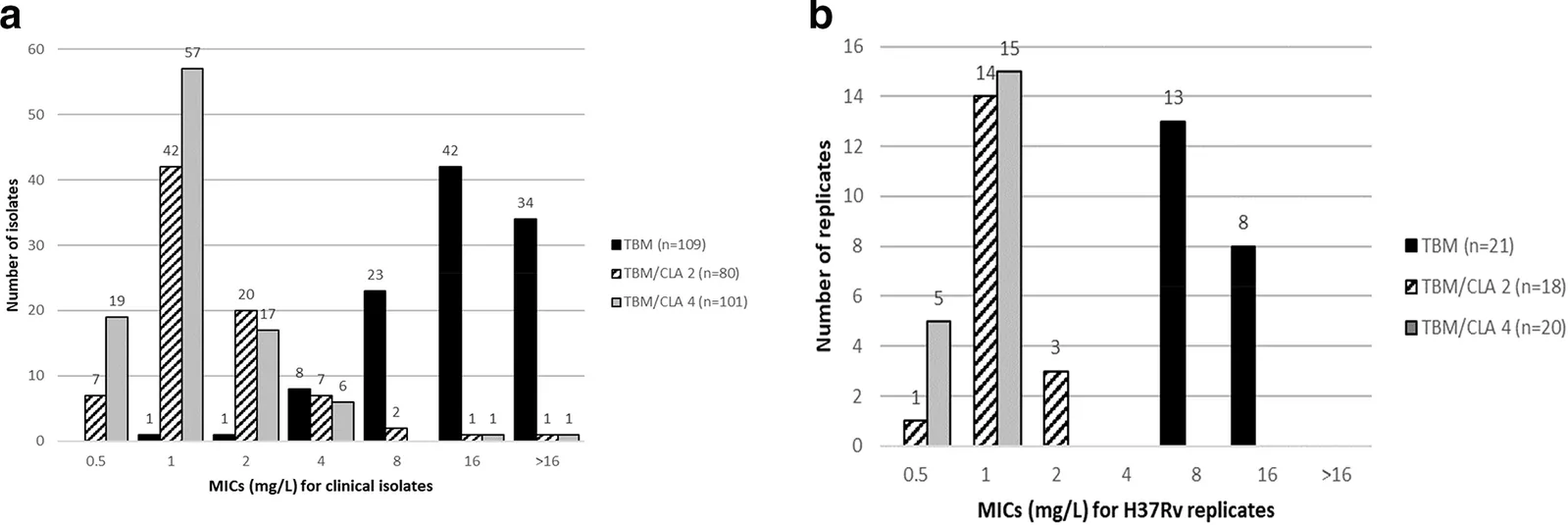
MICs for tebipenem alone or in combination with either 2 or 4 mg/L clavulanic acid. (**a**) 109 clinical isolates were tested once, and unusually low or high MICs are detailed in Table 1. (**b**) The H37Rv control strain was included in each batch of clinical isolates. CLA, clavulanic acid; TBM, tebipenem.

**Fig 2:**
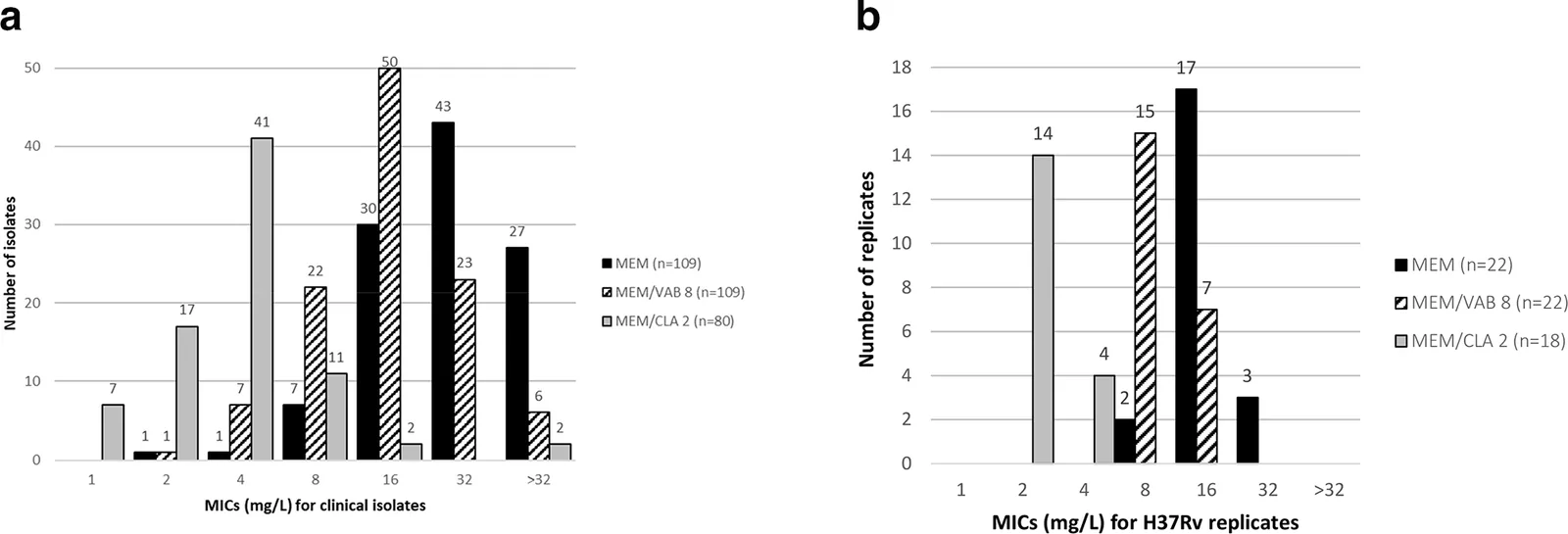
MICs for meropenem alone or in combination with either 8 mg/L vaborbactam or 2 mg/L clavulanic acid. (**a**) 109 clinical isolates were tested once, and unusually low or high MICs are detailed in Table 1. (**b**) The H37Rv control strain was included in each batch of clinical isolates. CLA, clavulanic acid; MEM, meropenem; VAB, vaborbactam.

MIC testing showed little technical variability given that the MICs of the H37Rv control strain used in each batch spanned between two and three dilutions (Fig. 1b and 2b). The modal MIC of tebipenem alone was 16 mg/L for clinical isolates, which was reduced 16-fold to 1 mg/L by the addition of clavulanic acid (Fig. 1a). Results were similar for 2 and 4 mg of clavulanic acid. The addition of a β-lactamase inhibitor also reduced the MICs of meropenem for clinical isolates with a larger reduction by clavulanic acid than vaborbactam (Fig. 2a). The modal MIC of meropenem-clavulanic acid (4 mg/L) was lower than that of meropenem alone (32 mg/L). The modal MIC of meropenem-vaborbactam (16 mg/L) was similar to the MIC of meropenem on its own (32 mg/L).

### Impact of lineage and MDR phenotype

Clinical isolates belonging to six different MTBC lineages were tested in the study. The majority (*n* = 40) were Euro-American strains of lineage 4, with 14 different sublineages represented. The remaining isolates belonged to lineage 1 (Indo-Oceanic, *n* = 14), lineage 2 (East-Asian Beijing, *n* = 26 and non-Beijing, *n* = 1), lineage 3 (East-African Indian, *n* = 19), lineage 5 (West Africa, *n* = 5), and lineage 7 (*n* = 4) (Table S2). The modal MICs of tebipenem-clavulanic acid, meropenem-vaborbactam, and meropenem-clavulanic acid did not differ between these six lineages. Similarly, there was no discernible difference in the MIC distribution for any of the tested antibiotics between MDR-TB isolates compared to the pan-susceptible isolates.

### Isolates with low MICs when tested without a β-lactamase inhibitor

Two isolates had low MICs for tebipenem (1 and 2 mg/L) and meropenem (2 and 4 mg/L) when tested individually, comparable to the MICs for these carbapenems in combination with a β-lactamase inhibitor (Table 1). A G207Stop mutation in *blaC* was found in SEA201800134 (Table 1), whereas no obvious genetic basis could be found in the susceptibility genes that we analyzed for SEA202000156 (Table S2). The *blaC* A49G mutation*,* associated with lower meropenem MICs in a study by Olivença et al. (21), did not have an apparent impact on the tebipenem or meropenem MICs in the only isolate (SEA201800390) that had this mutation (Table S2).

**TABLE 1:** Isolates with MICs at low- or high-end of distributions^*a*^

Isolate	MIC (mg/L) with BLI concentration in mg/L where applicable						Plausible explanation for usual MICs
	TBM	TBM/CLA 2	TBM/CLA 4	MEM	MEM/VAB 8	MEM/CLA 2	
SEA201800134	1^*b*^	1	1	2^*b*^	4	2	blaC G207Stop
SEA202000156	2^*b*^	0.5	0.5	4^*b*^	4	1	
SEA201500429	>16	**>16^*c*^**	**>16**	>32	>32	**>32**	
SEA202000213	>16		**16**	>32	>32		
SEA200700327	16	**16**		>32	>32	**>32**	Mixed infection with *M. intracellulare*

^*a*^ BLI, β-lactamase inhibitor; CLA, clavulanic acid; MEM, meropenem; TBM, tebipenem; VAB, vaborbactam.

^*b*^ Low MICs to either tebipenem alone (≤2 mg/L) or meropenem alone (≤4 mg/L).

^*c*^ High MICs to either tebipenem with clavulanic acid (≥16 mg/L) or meropenem with clavulanic acid (≥32 mg/L) are shown in bold.

### Isolates with high MICs when tested with a β-lactamase inhibitor

Three isolates had high MICs for tebipenem in combination with clavulanic acid (16 to >16 mg/L) or meropenem with clavulanic acid (>32 mg/L) (Table 1). A potential explanation could only be found for SEA200700327, which had also showed high meropenem-clavulanic acid MICs in an earlier study (Table S2) (17). This sample was found to be contaminated with *M. intracellulare* which might to be responsible for the high MICs (22). No other sample was found to be contaminated.

### Literature mutations implicated in higher MICs

**Fig 3:**
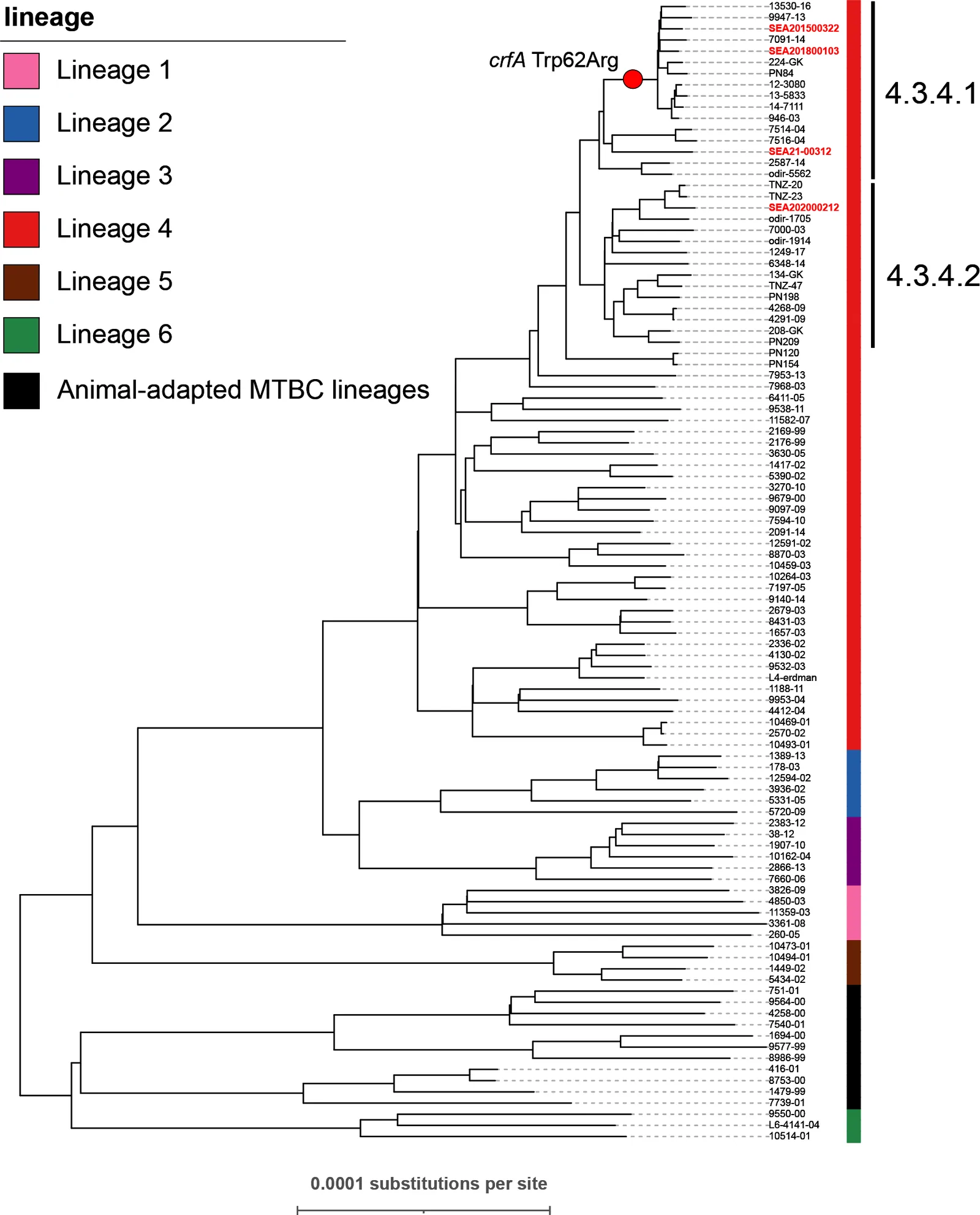
Phylogenetic relationship of crfA T62A and its correlation with MICs. Based on a phylogenetically diverse reference collection encompassing all major MTBC lineages, crfA T62A was shared by a subgroup of clinical lineage 4.3.4.1 isolates. The MICs of the two clinical lineage 4.3.4.1 isolates with crfA T62A and two clinical wild-type isolates (one from lineage 4.3.4.1 and the other from 4.3.4.2), which are highlighted in red, were measured. crfA T62A was found not to affect the MIC of any carbapenem (MICs presented in Table 2).

Based on three published studies (13, 17, 21), we had expected several isolates to show higher MICs to meropenem-clavulanic acid. First, SEA200600049 had an elevated meropenem-clavulanic acid MIC of 32 mg/L when previously tested on Middlebrook 7H10 (17), whereas this was not the case for any of the carbapenem-β-lactamase inhibitor combinations in this study, suggesting an error in the original testing or differences in methodology (17). Second, Kumar et al. found that the T62A change in *crfA* confers meropenem resistance in MTBC (13). When interrogating over 35,000 MTBC genomes sequenced at the Research Center Borstel, we found this mutation to be shared by a subgroup of lineage 4.3.4.1 isolates, but there was no impact on the MICs of respective strains in our study (Fig. 3; Table 2). Finally, we found that more than 80% of isolates in our study, irrespective of their MICs, harbored the six mutations previously associated with high meropenem MICs by Olivença et al., thereby calling the predictive power of this mutation into question (Table S2) (21).

**TABLE 2:** The crfA T62A mutation was shared by a subgroup of clinical lineage 4.3.4.1 isolates^*a*^

Isolate	*crfA* mutation	MIC (mg/L) with BLI concentration in mg/L where applicable					
		TBM	TBM/CLA 2	TBM/CLA 4	MEM	MEM/VAB 8	MEM/CLA 2
SEA201500322	T62A	4	1		8	4	2
SEA201800103	T62A	4	1		16	8	2
SEA21-00312	None	8	1		16	8	2
SEA202000212	None	8		1	16	16	

^*a*^ The MICs of the two clinical lineage 4.3.4.1 isolates with crfA T62A and two clinical wild-type isolates (one from lineage 4.3.4.1 and the other from 4.3.4.2) were measured. crfA T62A was found not to affect the MIC of any carbapenem. BLI, β-lactamase inhibitor; CLA, clavulanic acid; MEM, meropenem; TBM, tebipenem; VAB, vaborbactam.

## DISCUSSION

We studied the *in vitro* activity of different combinations of carbapenems and β-lactamase inhibitors, of which tebipenem-clavulanic acid showed the most promising effect. Clavulanic acid was a more potent β-lactamase inhibitor when combined with meropenem than vaborbactam, which supports the use of the currently recommended meropenem-clavulanic acid combination by WHO (19).

The lowest MICs in our study were seen for the orally available drugs tebipenem-clavulanic acid. Our finding of a modal MIC of 1 mg/L was in accordance with previous MIC studies on tebipenem-clavulanic acid for MTBC*,* both using a BMD methodology similar to ours. Li et al. reported a modal MIC of 1 mg/L for 122 clinical isolates, of which 47 isolates were drug-susceptible and 75 MDR/extensively drug-resistant (XDR)-TB isolates (15). Horita et al. found that the modal MIC of tebipenem-clavulanic acid to be 1 mg/L for drug-susceptible isolates (*n* = 20) and 0.25 mg/L for MDR/XDR-TB-isolates (*n* = 21) (14). Increasing the concentration of β-lactamase inhibitor above 4–5 mg/L has not been shown to affect the MIC of tebipenem combined with a β-lactamase inhibitor in multiple studies (14, 15). Currently, tebipenem is only used in Japan for the treatment of ear-nose-throat infections in children. A recently published large trial including 868 patients showed that tebipenem pivoxil hydrobromide was noninferior to intravenous ertapenem against complicated urinary tract infections (20). As tebipenem has the advantage of being an oral carbapenem with an existing safety profile, it may play a future role in the treatment of difficult-to-treat MDR-TB.

Meropenem-clavulanic acid showed greater *in vitro* activity compared to meropenem-vaborbactam (modal MICs 4 vs 16 mg/L, respectively). The modal MIC of meropenem-clavulanic acid in our study was 4 mg/L, which is in line with previous studies with modal MIC ranges of 2–8 mg/L (Middlebrook 7H9 broth) (15, 16, 18). Two studies reported lower modal MIC values of 0.5 mg/L (Middlebrook 7H9 broth) and 1 mg/L (7H10 solid agar) (14, 17). The latter 7H10 study used a clavulanic acid concentration of 64 mg/L compared to the EUCAST standard of 2 mg/L in this experiment. As clavulanic acid has no anti-tuberculous effect on its own (23 – 25), the differences in meropenem-clavulanic acid MICs from the studies are likely due to the choice of method. From a clinical perspective, meropenem-clavulanic acid is currently endorsed by the WHO as a treatment option for MDR-TB, mainly based on observational studies (26, 27). In addition, the recent phase 2A COMRADE study has shown early bactericidal activity, reducing colony-forming unit counts per milliliter of sputum of patients treated with meropenem-clavulanic acid (28).

To our knowledge, this is the first study evaluating meropenem in combination with vaborbactam for MTBC. Our modal MIC of 16 mg/L suggests limited anti-TB activity as the EUCAST non-species related breakpoint (29) for meropenem-vaborbactam is 8 mg/L, although it is unclear to what extent those breakpoints can be applied to MTBC, for which MICs are measured differently.

Whether the promising *in vitro* activity of tebipenem-clavulanic acid translates to similar or greater clinical efficacy compared with the currently WHO-endorsed meropenem-clavulanic acid requires further clinical studies. The COMRADE study showed poor tolerability for meropenem-clavulanic acid, with many gastrointestinal side-effects, and the authors called for an alternative oral carbapenem option (28). In addition to reducing patient discomfort, an oral carbapenem would shorten inpatient stays and reduce healthcare costs.

Our study also provided potential insights into mechanisms that confer either susceptibility or resistance to carbapenems and/or β-lactamase inhibitors that need to be explored in further studies. The G207Stop mutation might abolish BlaC function given that one third of the protein is no longer produced, including an essential residue required for its folding (30), which would explain the low carbapenem MICs for this isolate, even in the absence of β-lactamase inhibitors. Moreover, we found the T62A in the currently annotated *crfA* gene did not impact carbapenem MICs, despite strong evidence from Kumar et al. (13). Because of these contradictory findings, we investigated whether there was any evidence of expression of *crfA*. Of the three studies that mapped transcriptional start sites in *M. tuberculosis* (31 – 33), only Shell et al. found one 174 base pairs upstream of the putative start codon of *crfA*. Moreover, a translational site could not be found (34) and there was no evidence for transcription or translation coming from previously published RNA-seq (31, 32, 35) and ribo-seq studies (34, 35). This does not exclude the possibility that *crfA* is expressed under conditions not interrogated in these studies. Moreover, it is possible that *crfA* T62A only confers resistance in specific genetic backgrounds [e.g., epistasis that fully abolishes the effect of resistance mechanisms has been recently described for amikacin, bedaquiline, and clofazimine (36, 37)].

The main strength of this study was the large number of tested clinical isolates, including both pan-susceptible and MDR-TB isolates. Also, each batch of clinical isolates included the *M. tuberculosis* H37Rv ATCC 27294 reference strain as a technical control for MIC testing that demonstrated a good reproducibility of ±1 dilution steps (38, 39). Furthermore, we performed WGS of all isolates and analyzed multiple genes implicated in carbapenem susceptibility and resistance.

A limitation of our study was that we only used a single MIC method. Due to the intrinsic variability of MIC testing caused by biological and technical factors, including variations in the inoculum (38 – 40) retesting of all clinical isolates would have been preferable, particularly those with MICs at the lower or higher ends of the distribution. A further improvement of the experimental study design would be to add the β-lactamase inhibitor to the broth in the beginning of the experiment, thereby avoiding the addition of a very small volume of 2 µL to each well. Finally, because we did not include any XDR-TB isolates, our study did not provide any insight into the recently described phenomenon of collateral susceptibility whereby changes involved in resistance to other agents may render isolates more susceptible to β-lactam antibiotics (21, 41, 42).

In conclusion, tebipenem-clavulanic acid shows low MICs for MTBC, including MDR-TB isolates. The use of clavulanic acid in combination with meropenem resulted in a more pronounced reduction in MIC compared to vaborbactam. The potential use of the orally available drugs tebipenem-clavulanic acid for TB should be explored in clinical phase II drug trials.

## MATERIALS AND METHODS

The MICs of 109 MTBC clinical isolates (lineages 1–5 and 7) were determined using the EUCAST broth microdilution (BMD) reference method (43). The isolates included pan-susceptible (*n* = 69), pyrazinamide-resistant (*n* = 4), and MDR-TB-strains (*n* = 36), and WGS data were available for all isolates.

### Preparation of medium and anti-tuberculous agents

Middlebrook 7H9 medium (7H9), containing 10% OADC and 0.2% glycerol, was prepared according to the manufacturer’s instructions and stored in the fridge for maximum of 1 month. All anti-TB agents were dissolved with suitable solvent and then diluted to stock solutions in the desired concentrations (meropenem 0.25–32 mg/L and tebipenem 0.125–16 mg/L, Supplemental Table 1). Stock solutions were aliquoted in 0.2 mL vials and stored at −80°C for up to 12 months.

For tebipenem and meropenem, a 4× working solution was generated from an aliquot of previously prepared stock solution using two dilution steps in 7H9. A 100× working solution was made to give a fixed concentration of 8 mg/L for vaborbactam and 2 or 4 mg/L for clavulanic acid in the microtiter plate when adding a volume of 2 µL in each well, according to the plate layout (Fig. S1a through f). The concentration of 4 mg/L of clavulanic acid was initially chosen based on Horita et al. (14). However, since the EUCAST standard for susceptibility testing of clavulanic acid is a fixed concentration of 2 mg/L, a subset of 80 isolates was also tested using this concentration (29). Fresh working solutions for each substance were made on each day of testing.

### Preparation of microtiter plates

For the BMD, sterile U-bottom-shaped 96-well polystyrene microtiter plates with an untreated surface were used. The plates were inoculated according to the plate outline (Fig. S1a through f). The peripheral wells were inoculated with 200 µL sterile water to minimize evaporation. The rest of the wells were inoculated with 100 µL 7H9 for the plates, where only one drug was tested or 98 µL 7H9 for the plates where a combination of two drugs was tested. In the plates containing only one drug, 100 µL of the 4× working solution was added to the left row (with the highest drug concentration) and a multi-channel pipette was used to make 1:2 dilutions from the highest concentration row to the following rows and the last 100 µL from the last row was discarded. For plates where a drug combination was tested, the serial dilution step was followed by adding 2 µL of either vaborbactam or clavulanic acid to each drug-containing well, according to the plate layout.

### Preparation of mycobacterial inoculum and inoculum dilutions

Fresh cultures of MTBC grown on Löwenstein–Jensen media were used within 2 weeks from the first visible growth. The *M. tuberculosis* H37Rv ATCC 27294 reference strain was included as an internal control in each test round (*n* = 22). Approximately three to four 1 µL loops of growth were added to a glass tube containing five to six glass beads with a size of 3 mm, the tube was vortexed for 2 min, followed by adding of 5 mL sterile water and then another 2 min of mixing using vortex. The bacterial suspension was then left to sediment for 30 min. After sedimentation, the inoculum was transferred to a new dry glass tube and the turbidity was adjusted to McFarland 0.5 using a nephelometer. Dilutions of the bacterial suspension were performed by four serial dilution steps where 1 mL of suspension was added to 9 mL 7H9 and vortexed. This process was repeated three times, resulting in four dilutions of 10^−1^, 10^−2^, 10^−3^, and 10^−4^.

### Inoculum and incubation of the plates

The previously prepared microtiter plates were inoculated with 100 µL of the 10^−2^ dilution in all the drug-containing wells as well as the undiluted growth control. About 100 µL of the 10^−4^ dilution was used for the 1:100 growth control. A total of six isolates, including a H37Rv reference strain, could be tested in one plate. The inoculated microtiter plates were placed in plastic bags, with a maximum of three plates on top of each other and incubated at 36°C for 7–21 days.

### Reading and interpretation of results

The plates were read on days 7, 10 (9–11), 14, and 21, using an inverted mirror. The MIC was defined as the lowest drug-containing concentration that did not show visible growth on the first day that growth was detected in both positive growth controls (i.e., the undiluted and 1:100 growth control).

### Whole-genome sequencing

DNA was extracted from MTBC strains growing on Löwenstein-Jensen medium with the QIAamp Mini DNA Kit (Qiagen, Hilden, Germany) (44) or a chloroform/cetrimonium bromide (N-cetyl-N,N,N-trimethyl ammoniumbromide)-based protocol (45). The vast majority of the samples (*n* = 106) were sequenced with an Ion Torrent S5 XL instrument (Thermo Fisher Scientific Inc, Waltham, MA, USA), according to the manufacturer’s instructions (read length, approximately 300 base pairs). The three remaining samples had previously been sequenced on an Illumina platform (46). All samples were analyzed in the same bioinformatic pipeline using the H37Rv NC_000962.3 genome as the reference genome. The variants obtained were subsequently filtered according to the following parameters: minimum coverage: 10; minimum count: 2; minimum frequency for single-nucleotide polymorphisms: 10%; minimum frequency for insertions and deletions: 80%) (CLC Genomics Workbench 21.0.4, Qiagen, Hilden, Germany). MTBC lineages were assigned according to Coll et al. (47). *blaC*, *crfA* (position 2,718,665–2,719,060), *murG*, *murD*, *ftsH*, *ftsK*, *ponA1*, *pbpB*, *chiZ*, and six mutations that were associated with elevated meropenem MICs by Olivença et al. were analyzed (13, 21). *crfA* I63S was assumed not to be relevant as it was shared by all isolates in this study and was, consequently, not shown in Fig. 3. Contamination by other bacteria was identified using Kraken2 (48). To place isolates with the mutation *crfA* T62A into a broader phylogenetic context, we employed MTBseq with default parameters for variant calling for a comparative genomic analysis including publicly available data sets of major MTBC lineages (49). The resulting concatenated sequence alignment was used to calculate a maximum likelihood tree using iqtree, excluding invariant sites, and with ascertainment bias correction (50).

## Data Availability

The accessions for all raw sequencing reads are presented in Table S2.

## SUPPLEMENTAL MATERIAL

Table S1 and Figure S1 spectrum.01781-23-s0001.docx Preparation of test concentrations and plate layout BMD.

Table S2 spectrum.01781-23-s0002.xlsx Raw data of minimum inhibitory concentrations and WGS.

ASM does not own the copyrights to Supplemental Material that may be linked to, or accessed through, an article. The authors have granted ASM a non-exclusive, world-wide license to publish the Supplemental Material files. Please contact the corresponding author directly for reuse.
