# Supplementary material for: In vitro activity of new combinations of β-lactam and β-lactamase inhibitors against the Mycobacterium tuberculosis complex
Source: Microbiol Spectr. 2023 Sep 22;11(5):e01781-23. doi: 10.1128/spectrum.01781-23 (PMC10580993; doi:10.1128/spectrum.01781-23)
Supplement: Table S1 and Figure S1 — Preparation of test concentrations and plate layout BMD. [file spectrum.01781-23-s0001.docx]

**Supplemental Table 1.** Preparations of test concentrations.

| **Β-lactam antibiotic / β-lactamase inhibitor** | **Solvent** | **Stock solution (mg/L)** | **Dilution 1 7H9 without OADC** | **Dilution 2**  **7H9 with OADC** | **Working solution (mg/L)** | **Concentration range (mg/L)** |
| --- | --- | --- | --- | --- | --- | --- |
| **Tebipenem** | H_2_O | 10,240 | 1:20 | 1:8 | 64 | 0.125 -16 |
| **Potassium clavulanate 1:1** | H_2_O | 10,800 | 1:9 | 1:3 | 400 | 4 |
| **Meropenem** | H_2_O | 10,240 | 1:20 | 1:4 | 128 | 0.25-32 |
| **Vaborbactam** | DMSO* | 9,600 | 1:6 | 1:2 | 800 | 8 |
| **Isoniazid** | H_2_O | 10,240 | 1:64 | 1:40 | 4 | 0.008-1 |

Abbreviations: H_2_O: water, DMSO: dimethyl sulfoxide

**Supplemental Figure 1 a-f.** Plate layout of 96-well polystyrene microtiter plates for broth microdilution-MIC testing of Mycobacterium tuberculosis complex for

1. Meropenem (MEM)
2. Tebipenem (TBM)
3. Meropenem-vaborbactam (MEM-VAB), vaborbactam concentration 8 mg/L
4. Meropenem-clavulanic acid (MEM-CLA), clavulanic acid 2 mg/L
5. Tebipenem-clavulanic acid (TBM-CLA), clavulanic acid concentration 2 mg/L
6. Tebipenem-clavulanic acid (TBM-CLA), clavulanic acid concentration 4 mg/L
